# Scale up of anaesthesia services in underserved rural Tanzania

**DOI:** 10.1186/s12913-023-09963-x

**Published:** 2023-09-18

**Authors:** E. Kweyamba, AS Nyamtema, JC LeBlanc, A. Shayo, RB George, H. Scott, O. Kilume, J. Bulemela, Z. Abel, G. Mtey

**Affiliations:** 1 Tanzanian Training Centre for International Health, Ifakara, Tanzania; 2 St. Francis University College for Health and Allied Sciences, Ifakara, Tanzania; 3 Pediatrics, Community Health and Epidemiology and Psychiatry, Dalhousie University, Dalhousie, Canada; 4 Mount Sinai Hospital, University of Toronto, Toronto, ON Canada; 5 Department of Obstetrics and Gynaecology, Dalhousie University, Dalhousie, Canada

**Keywords:** Quality and safety of anaesthesia, Anaesthesia in obstetrics, Non-physician anaesthesia providers, Clinical audit

## Abstract

**Background:**

Because of critical shortage of physician anaesthesiologists, the government of Tanzania adopted a task shifting strategy for provision of anaesthesia services. This paper describes the results of an operational study designed to increase the number of anaesthesia providers for emergency obstetric surgeries in order to reduce maternal and perinatal mortality in underserved rural Tanzania.

**Methods:**

In 2016 a before-after cohort study was conducted in seven health centres in rural Tanzania. Five health centres received an intervention and two were selected to track secular trends (control group). Ten associate clinicians, i.e. assistant medical officers, clinical officers, and nurse midwives, from five health centres were trained in anaesthesia skills for emergency obstetric surgeries for three months followed by quarterly supportive supervision, mentoring and teleconsultation to reinforce skills. Primary and secondary outcome measures included Caesarean delivery (CD) rate, quality and safety of anaesthesia, and uptake of the educational program for anaesthesia.

**Results:**

Out of the 2,179 CDs performed in the intervention facilities from 2016 to 2019, two women died from complications of anaesthesia. The risk of death from anaesthetic complications was 0.9 per 1000 CD (95% CI 0.1–3.3. The risk of death was not established in the control group because of inadequate documentation and records keeping. The proportion of CD performed under spinal anaesthesia in intervention facilities doubled from 28% (60/214 with 95% CI 22–35) at baseline (July 2014 – June 2016) to 57% (558/971 with 95% CI of 54–61) in year three (July 2018 - June 2019), while in the control group increased by only 40% from 19% (92/475 with 95% CI of 16–23) at baseline and 27% (68/251 with 95% CI of 22–33) in year three. In 2020I, this educational training program was then adopted by the government with minor content changes and increasing duration of training to six months.

**Conclusions:**

This three month educational training program for associate clinicians in anaesthesia, complemented by supportive supervision, can increase the CD rate to one that fills the “unmet need” and the proportion of operations performed under spinal anaesthesia, the gold standard technique for CD. The program can be used to meet the urgent demand for anaesthesia services in other underserved areas in Africa.

## Introduction

Anaesthesia is usually required for the surgical management of the leading causes of maternal and perinatal deaths and disability (obstetric hemorrhage, abortion, eclampsia and obstructed labour). Based on a 2015/16 survey, the maternal mortality ratio in Tanzania was 556 deaths per 100,000 live births [1]. 93% of all reported maternal deaths in 2019 occurred within health facilities from complications of surgically manageable conditions including abortions and uterine rupture which contributed to 2.6% and 6.4% of the deaths respectively [2]. One of the factors crippling provision of obstetric surgical services is inadequate number of trained anaesthesia providers. Because of the low density of physician anaesthesia providers, the government of Tanzania after independence in early 1960s adopted a task shifting strategy for provision of anaesthesia services. Despite using Non-Physician Anaesthesia Providers (NPAPs) under this task shifting policy, there continues to be a huge unmet demand in the country. In 2012 the density of anaesthesia providers (including NPAPs) was 0.15 per 100,000 persons [3, 4]. During the time of writing this paper there were only 36 physician anaesthesia providers in Tanzania with estimated population of 60 million, which is equivalent to 0.06 physician anaesthesia providers per 100,000 population [5]. The density of anaesthesia providers in Tanzania is far lower than the minimum requirement of 4–5 per 100,000 people advocated for by World Federation of Societies of Anaesthesiologists (WFSA) [6].

Given a critical shortage of physician anaesthesia providers at present as well as a shortage of training positions in Tanzanian medical schools, it will take decades to train enough physician anaesthesia providers to staff every health centre (HC) and district hospital. Until then, non-physician clinicians will remain the backbone of anaesthesia in Tanzania. In 2014, the Accessing Safe Deliveries in Tanzania (ASDIT), one of the projects supported by the Innovating for Maternal and Child Health in Africa (IMCHA) initiative, was designed to study strategies to increase the number of care providers able to provide safe anaesthesia in order to support the National Road Map Strategic Plan for 2016–2020 to expand the number of health centres providing comprehensive emergency obstetric and neonatal care (CEmONC) from 12% to 2015 to 50% by 2020 [7]. From 2016 to 2019 the project designed and implemented an educational training program for anaesthesia using associate clinicians (assistant medical officers, clinical officers and nurse midwives). The trained associate clinicians were selected by the Council Health Management Teams of respective districts. Implementation of this program was accompanied by the introduction and strengthening of CEmONC services in five health centres located in underserved rural areas in Morogoro region through training and support of obstetrical care providers. This paper provides results of implementing an educational training curriculum for anaesthesia for CEmONC services and how the objective was achieved in underserved rural Tanzania.

## Methods

### Study settings and design

A before-after cohort study design was conducted in seven health centres in Tanzania. The Tanzania health system starts, in ascending order, from dispensaries, health centres, district hospitals, regional hospitals, zonal referral hospitals and the national hospitals. The study was conducted in five intervention and two control health centres from four district councils (DCs) of Morogoro region. The five intervention health centres were Gairo in Gairo DC, St. Joseph in Kilosa DC, Ngerengere in Morogoro DC, and Melela and Kibati in Mvomero DC. The two control health centres were Mkamba and Mlimba from Kilombero DC. Intervention centres were chosen to reflect the diversity of funding and management of HCs in Tanzania (e.g., public vs. non-governmental organization) and are therefore not meant to be individual samples from the same population of HC. Similarly, the control centres were chosen to track secular trends, i.e., changes in epidemiology and health services practices that occurred independently of the ASDIT intervention. Like the intervention centres, they are not samples from a single underlying population of HCs and therefore caution must be used in making statistical comparisons between intervention and control centres. The distance to nearest referral hospitals for the five intervention health centres ranged from 40 to 93 km and represented realistic travel times of one to several hours. The two control health centres were 70 to 150 km from the referral hospital.

### Intervention

Two experienced personnel (nurse-midwives and/or clinical officers) from each of the five intervention HCs were enrolled in an existing three month NPAPs training program at the Tanzanian Training Centre for International Health (TTCIH) and St. Francis Referral Hospital in Ifakara. The training was run by an anaesthesiologist from Muhimbili National Hospital (MNH) and experienced Assistant Medical Officer anaesthesiologists from TTCIH, St. Francis Referral Hospital and the Kilimanjaro Christian Medical Centre- AMO Anaesthesia School. They received didactic and practical instruction in the provision of general and spinal anaesthesia for elective and emergency obstetric care as well as instruction on the care of sick and premature newborns. Prior to our intervention NPAP were rare in these health centres.

Training methods included, lectures, simulations, skills demonstration in clinical skills laboratory and supervised services on actual patients. “Hands on” training was emphasized and trainees spent at least 70% of their time in the supervised provision of pre-operative, intra-operative and post-operative care to patients. They took part in both elective and emergency operations for general surgery, obstetrics and gynaecology. The trainees took night calls under the supervision of more experienced hospital staff. Clinical practices were designed to strengthen trainees’ proficiency, self-confidence, problem solving, clinical decision making and reflective thinking skills. The acquired competencies were objectively evaluated through various methods including pre-test and post-test knowledge evaluations, observed structured clinical evaluations (OSCE), and direct observation. Each trainee kept a procedure logbook to track their exposures and learnings.In addition to the anaesthesia training program there were other measures implemented to strengthen CEmONC at these sites including training and post-training coaching and mentorship of other health care providers on comprehensive emergency obstetric and newborn care, e-learning, supported essential supplies and equipment and minor renovations to some facilities.

### Post-training follow-up

Post-training knowledge and skills strengthening was done through E-learning modules that were developed as part of a collaboration between the TTCIH team, anaesthesiologists from both MNH and Dalhousie University and deployed on desktop computers with on-site quarterly supportive supervision and mentorship visits.These visits included chart review auditing of anaesthetic services and outcomes. The supervisors were a team of anaesthesia experts (Anaesthesiologists and experienced skilled NPAPs) from MNH, Dalhousie University, TTCIH, St. Francis Referral Hospital and members of Council Health Management Team or Regional Health Management Team for the respective districts. In addition to supportive supervision, team members functioned as mentors for the graduates. A 24-hour tele-consultation service was established so that the graduates could call any time of the day or night for help. All worrisome cases that were beyond the skill level of the graduates were referred to district hospitals. The E-learning modules developed for desktop computers were migrated to the Moodle platform on smartphones so that they would be more accessible to graduates.

Project planning and implementation involved the Government decision makers at the beginning and at all levels of authority, from the level of Ministry of Health down to the districts. The project team included the project co-PI decision maker (GM) who was the Regional Medical Officer of Morogoro region. The project conducted progress and feedback meetings every six months, which involved decision markers at regional, district and facility levels. There were also annual meetings for national key stakeholders including the Ministry of Health and President’s Office Regional Administration and Local Government (PORALG).

### Data collection

During supportive supervision and mentorship visits, the project team reviewed the data on types of obstetric surgeries, choice of anaesthetic techniques, appropriateness (i.e., the case aligned with site resources), and outcome of anaesthesia. These data were collected from anaesthetic charts, patient case files, surgical procedure logbook, and Health Information Management System (HIMS) books. All maternal deaths were audited in order to establish cause of death and the role of anaesthesia. Audit of maternal deaths in the control HCs could not be completed because of inadequate documentation and records keeping Anonomyous data entry was done via tablets using the CommCare application.

### Assessment of safety of anaesthesia

In the past four decades spinal anaesthesia has been adopted as the safest and hence most frequently administered type of anaesthesia for CD in high income countries such as the United States [8]. Safety of anaesthesia in this study was assessed using two indicators: appropriateness in the selection of anaesthetic technique, i.e. a greater utilization of spinal anaesthesia, and a patient outcome measure, specifically maternal mortality (Maternal death occurring during or within 24 h of anaesthesia or after the failure of women to regain consciousness after anaesthesia). Because of suboptimal documentation, complications of anaesthesia were not included in this paper. Use of morbidity measures for quality and safety assessment has substantially limited sensitivity and specificity because morbid incidents largely rely on the willingness of staff members to report them [9].

### Strategies for uptake

Strategies for uptake of the educational program involved continuous engagement of multiple stakeholders including decision-makers at the district and regional levels, policy-makers at the Ministry of Health and President’s Office Regional Administration and Local Government, and the Society of Anaesthesiologists of Tanzania (SATA). Engagement strategies included regular meetings and workshops for project implementation updates.

### Data analysis

Data were extracted from the CommCare application server into Microsoft Excel and analyzed using Stata, version 15. The Chi-squared test was used to determine the statistical differences of the proportions of CDs performed under spinal anaesthesia between and within the intervention and control health centres. The level of significance was set at p-value at 0.05.

## Results

Provision of obstetric surgeries and anaesthetic services were strengthened at St Joseph, Gairo and Melela HCs, and were introduced at Ngerengere and Kibati HCs. The intervention HCs experienced a substantial increase in the number of women coming to the HC for delivery care after these centres began providing obstetric surgeries (Table 1). The number of all deliveries in the control and intervention HCs were 5,709 and 4,392 at baseline (July 2014 – June 2016) compared to 11,233 and 12,918 respectively during the intervention period (July 2016 – June 2019). As opposed to the control HCs, the CD rate increased significantly in the four intervention HCs. The overall CD rate in the intervention HCs increased from 12.7% (95% CI 11.8–13.7) at baseline to 16.9% (95% CI 16.2–17.5) during the intervention period.

**Table 1:** Caesarean delivery rates in the intervention and control health centres before and after the intervention

	Baseline period			Intervention period		
	All deliveries	Caesarean deliveries	CD rate (95% CI)	All deliveries	Caesarean deliveries	CD rate (95% CI)
*Intervention HCs*						
St. Joseph	1,155	341	29.5 (26.9–32.2)	4,656	957	20.6 (19.4–21.7)
Ngerengere	618	0	0 (0.6)*	1,202	116	9.6 (8.0–11.5)
Kibati	494	0	0 (0.7)*	1,206	97	8.0 (6.6–9.7)
Melela	410	4	1 (0.3–2.5)	921	149	16.2 (13.9–18.7)
Gairo	1,715	214	12.5 (11.0–14.1)	4,933	860	17.4 (16.4–18.5)
Overall interv. HCs	4,392	559	12.7 (11.8–13.7)	12,918	2,179	16.9 (16.2–17.5)
*Control HCs*						
Mkamba	1,759	111	6.3 (5.2–7.5)	3,018	23	0.8 (0.5–1.1)
Mlimba	3,950	391	9.9 (9.0–10.9)	8,215	946	11.5 (10.8–12.2)
Overall control HCs	5,709	502	8.8 (8.1–9.6)	11,233	969	8.6 (8.1–9.2)

Note: Baseline = July 2014 – June 2016; intervention period = July 2016 – June 2019; HCs = health centres; CD = caesarean deliveries; (*) one-sided, 97.5% confidence interval

### Choice of anaesthetic techniques

**Fig. 1:**
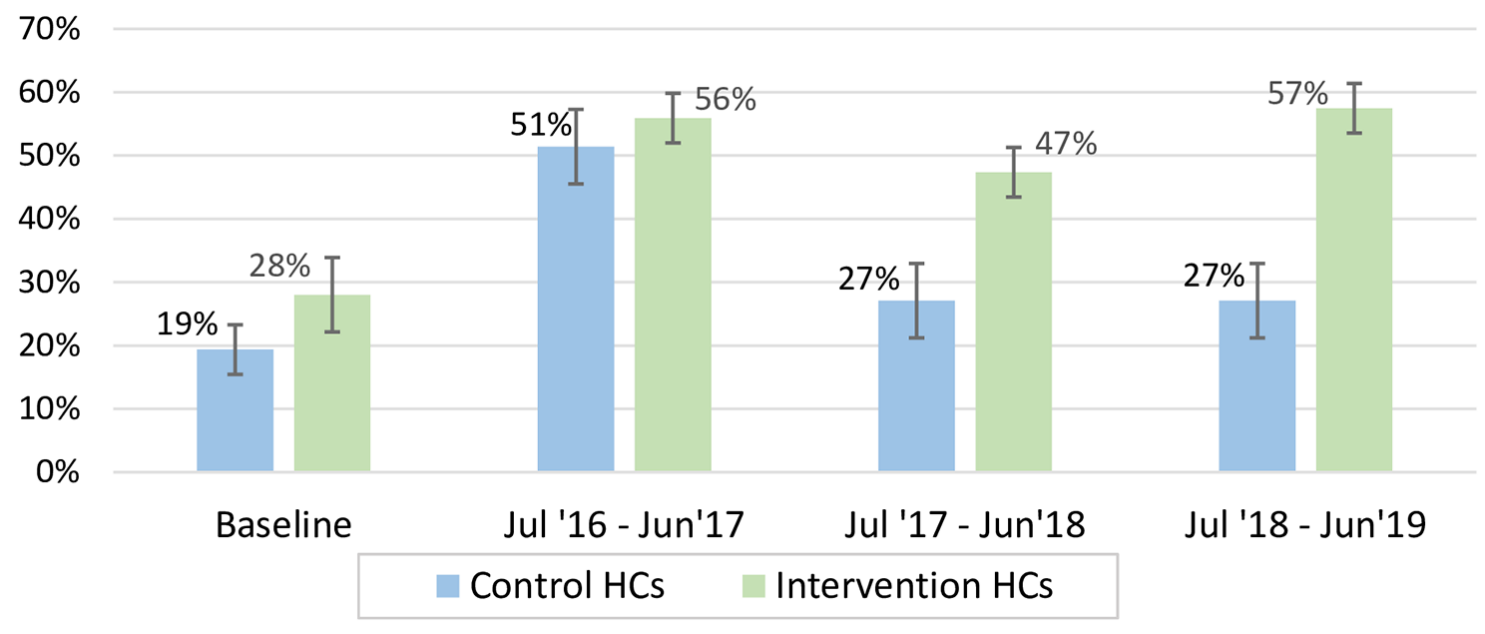
The annual proportions of caesarean deliveries performed under spinal anaesthesia in the intervention and control health centres. Note: Baseline = July 2014 – June 2016; HCs = health centers

All intervention and control HCs provided spinal or ketamine anaesthesia for all obstetric surgeries. The proportion of CDs performed under spinal anaesthesia in intervention HCs doubled from 28% (60 out of 214 with 95% CI 22–35) at baseline (July 2014 – June 2016) to 57% (558 out of 971 with 95% CI of 54–61) in year three (July 2018 - June 2019), while in control group increased by only 40% from 19% (92 out of 475 with 95% CI of 16–23) at baseline to 27% (68 out of 251 with 95% CI of 22–33) in year three (Fig. 1).

### Quality and safety of anaesthesia

During the intervention period a total of 516 women had obstetric haemorrhage (Table 2). Of these 389 (75.3%) were reported in the intervention HCs. The leading cause was postpartum haemorrhage due to uterine atony, which contributed to 55% (283) of all cases reported in the intervention and control health centres.

**Table 2:** Causes of obstetric haemorrhage from the intervention and control health centres during the intervention period

	Intervention HCs n (%)	Control HCs n (%)	Total n (%)
Ectopic pregnancy	40 (10.3)	7 (5.5)	47 (9.1)
Antepartum haemorrhage	72 (18.5)	36 (28.3)	108 (20.9)
Uterine rupture	53 (13.6)	12 (9.4)	65 (12.6)
Postpartum haemorrhage (uterine atony)	214 (55.0)	69 (54.3)	283 (54.8)
Cervical tear	5 (1.3)	2 (1.6)	7 (1.4)
Retained products of conception	5 (1.3)	1 (0.8)	6 (1.2)
*Total*	*389 (100)*	*127 (100)*	*516 (100)*

Note: Complications of abortion were not included in this analysis

Appropriateness in the selection of type of anaesthesia: although active haemorrhage is a relative contraindication for spinal anaesthesia, from July 2016 to June 2019, in the intervention HCs 19.4% (7/36) of women with a ruptured uterus, 10% (4/40) with ectopic pregnancies and 40.0% (8/20) with indication of antepartum hemorrhage received spinal anaesthesia. Prior to July 2016 in the intervention group and prior to July 2019 in control group data were insurfficient to analyze. Because of inadequate documentation, type of anaesthesia was identified in only 36 cases (67.9%) of uterine rupture in the intervention health centres.

Outcome of anaesthesia: Out of the 2,179 CDs performed in the intervention facilities during the intervention period two women died from complications of anaesthesia. The risk of a woman dying from complications of anaesthesia (maternal death occurring during or within 24 h of anaesthesia or after the failure of women to regain consciousness after anaesthesia*)* in the intervention HCs was 0.9 per 1,000 CD (95% CI 0.1 to 3.3). Maternal deaths in the control facilities were not audited because of the absence of case files. In addition, the anaesthetic logbooks in the control HCs were not designed to capture surgical and anaesthetic complications.

### Uptake of educational program for anaesthesia

Anecdotally, HC staff appreciated the supportive supervision visits and frequently used the 24-hour phone access for difficult cases. The findings from this project reinforced government efforts to develop a standardized competency based curriculum for a short course on anaesthesia with a few improvements, including increasing duration of training from three to six months. The uptake was accelerated by increased demand for anaesthesia providers in the country that was created between 2015 and 2019 when the government constructed and upgraded 419 health facilities (consisting of 350 health centres and 69 district council hospitals) for comprehensive emergency obstetric and newborn care services [10]. In this case anaesthetic personnel are involved in regional and districts teams when conducting onsite coaching and mentorship to health care providers on emergency obstetrics and newborn care. This is currently taking place in Morogoro region.

## Discussion

Delivery of the three months curriculum for anaesthesia enabled the introduction of emergency obstetric surgeries at health centres in underserved rural areas in Tanzania. The number of CDs done under spinal anaesthesia increased substantially.

### Choice of anaesthetic techniques

The choice of anaesthetic techniques for CD depends on several factors including physiological presentation of the patient, experience level of the practitioner, availability of drugs, and equipment, among others. In this study the use of spinal anaesthesia for CD increased over time in the intervention group and for most cases was preferred over ketamine-based monitored anaesthesia care. Nevertheless, the use of spinal anaesthesia for CD, while higher than during the pre-intervention period, did not continue to increase. This was due largely to irregular supply of spinal anaesthetic agents and some staff being more comfortable with ketamine-based monitored anaesthesia care than with placing a spinal needle. Following implementation of a similar three month training in anaesthesia, the study conducted in rural health centres of Kigoma region in Tanzania showed increased use of spinal anaesthesia from 10% during baseline to 64% during the intervention period [11]. Although both general and spinal anaesthesia are safe and effective for CDs, spinal anaesthesia is favoured as the better choice. Compared to general anaesthesia, spinal anaesthesia is less likely to cause respiratory depression, causes a smaller risk of drug toxicity for both mother and baby, is followed by higher 1 and 5 min Apgar scores and blood loss is less. Spinal anaesthesia also facilitates earlier contact with the baby and initiation of breastfeeding [12–14]. Although spinal anaesthesia is safe and effective, it can have associated complications such as hypotension, post-dural puncture headache, and nerve damage. Findings from this study suggest procurement improvement is necessary in order to improve anaesthetic practices. Ongoing education in the choice of anaesthetic techniques along with patient education should aid in accelerating the use of spinal anaesthesia.

### Appropriateness of selection of anaesthetic techniques

The proper choice of anaesthetic technique, skills and experience of anaesthetics are important determinants to achieve safe anaesthesia in obstetric surgeries [15]. The use of spinal anaesthesia in 19.4% of women with ruptured uterus and 10% of ectopic pregnancies was one of the gaps demonstrated by the outputs of the educational program. ‘The reason for selection of an inappropriate type of anaesthesia could be partly explained by the fact that these diagnoses were established intra-operatively rather than pre-operatively. These findings suggest the need to improve clinical training of both anaesthesia and obstetrics providers in the recognition and preoperative evaluation and management of abdominal hemorrhage via continuous strengthening of knowledge and skills among non-physician anaesthesia providers.

### Safety of anaesthesia

Out of 2,179 CDs performed during the intervention period, two deaths were attributed to complications of anaesthesia. The risk of woman dying from complications of anaesthesia in project-supported health facilities was 1 per 1,000 CDs (95% CI 0.1 to 3.3) which was in the same range as that reported from rural health centres in Kigoma region (0.5 per 1,000 CD) in 2016, Zimbabwe (2.1 per 1,000 CD) in 2005, Nigeria (2.5–3.7 per 1,000 CD) in 2008 and in low and middle-income countries (1.2 per 1,000 procedures) [11, 16–18]. In the absence of anaesthetic services more women might have lost their lives and/ or their babies. Considering that many areas in Tanzania suffer under similar constraints, it is possible that expanding the educational programme to other regions might improve the provision of anaesthetic services especially for pregnant mothers and their babies. Looking at a broader picture, the estimate of the risk in these health facilities is significantly higher than those reported in high-income countries such as the USA, where the case fatality rate from general and regional anaesthesia for CD were 6·5 and 3·8 per million anaesthetics, respectively [19]. Since the majority of causes of anaesthesia-attributed deaths such as complications of airway management, pulmonary aspiration, and hypotension are preventable with appropriate training and resources to facilitate spinal anaesthesia, efforts should be made to improve skills and essential equipment and supplies.

### Uptake of educational training program

Though the government of Tanzania had initiated upgrading of the public health centres to provide comprehensive emergency obstetrics and newborn care that includes CD, there was a challenge with respect to human resources available to provide anaesthesia services. The findings from the project strengthened the government decision to use health care providers who have undergone a short course on anaesthesia. The government improved the curriculum that was used in the project with inputs from stakeholders that included extension of training duration from three to six months, standardizing the curriculum and adoption as National documents instead of Institutional. This has given the opportunity for five institutions in Tanzania to conduct this type of training for non-physician clinicians. These institutions are Muhimbili National Hospital, Muhimbili Orthopedic Institute, Kilimanjaro Christian Medical Centre, Mbeya Consultant Hospital and Tanzanian Training Centre for International Health. Various factors contributed to the success of the project uptake including choice of an intervention that aligned with government priorities, and engagement of policy and decision makers throughout the project period. Uptake was also ascribed to involvement of SATA and the academic departments of anaesthesia at the schools of anaesthesia in Tanzania. Involvement of decision maker in project implementation team at level co-principal investigator was another innovation that accelerated uptake.

### Limitations of the study

We were unable to collect information on maternal deaths and anaesthetic complications in control health centres because of a lack of files and anaesthetic logbooks were not designed to capture surgical and anaesthetic complications. Because of inadequate documentation, type of anaesthesia was identified in only some patients with uterine rupture in the intervention health centres. Nevertheless, important quantitative information was gained from each HC studied.

## Conclusions

The three months training program for clinical officers and nurse-midwives in anaesthesia, complemented by supportive supervision and mentorship program can increase the CD rate and the proportion of operations performed under spinal anaesthesia, the gold standard technique for CD, to save lives of mothers and babies in underserved rural Tanzania. These results provide a body of evidence that more mothers can have access to obstetric anaesthesia services with available mid-level health care providers in resource limited settings.

## Data Availability

The datasets generated and/or analysed during the current study are not publicly available due to government restrictions on sharing data but are available from the corresponding author on reasonable request.
